# Systemic Inflammation and Myocardial Repolarization Heterogeneity in Heart Failure and Obstructive Sleep Apnea: Impact on Arrhythmic Risk

**DOI:** 10.3390/medicina61091674

**Published:** 2025-09-15

**Authors:** Emirhan Çakır, Uğur Özkan, İlker Yılmam

**Affiliations:** 1 Department of Cardiology, School of Medicine, Trakya University, 22030 Edirne, Turkey; emir.cakir05@gmail.com; 2 Department of Pulmonology, School of Medicine, Trakya University, 22030 Edirne, Turkey

**Keywords:** apnea/hypopnea index, obstructive sleep apnea, heart failure, myocardial repolarization heterogeneity, systemic immune-inflammation index, ventricular arrhythmias

## Abstract

*Background and Objectives*: Obstructive sleep apnea syndrome (OSAS) and heart failure (HF) frequently coexist, amplifying cardiovascular risk through mechanisms involving chronic inflammation and autonomic dysfunction. This study investigates the impact of systemic inflammation, measured by the systemic immune-inflammation index (SII), and OSAS severity, assessed by the apnea–hypopnea index (AHI), on myocardial repolarization heterogeneity in patients with both conditions. *Materials and Methods*: In this retrospective study, 160 patients with HF and polysomnography-confirmed OSAS (AHI ≥ 5 events/h) were evaluated between January 2018 and November 2024. Patients were stratified by QT dispersion (QTd < 40 ms vs. ≥40 ms) to assess electrical heterogeneity. SII was calculated from neutrophil, platelet, and lymphocyte counts, and electrocardiographic markers (QTd, frontal QRS-T angle, T wave peak-to-end interval [TPEI]) were measured. Logistic regression and receiver operating characteristic (ROC) analyses were used to identify predictors of repolarization heterogeneity and ventricular arrhythmias. *Results*: Patients with QTd ≥ 40 ms (*n* = 78) exhibited higher SII (*p* < 0.001) and AHI (*p* < 0.001) compared to those with QTd < 40 ms (*n* = 82). SII and AHI independently predicted increased QTd in multivariate analysis (*p* = 0.01 and *p* < 0.001, respectively). ROC analysis identified SII ≥ 625.4 (sensitivity 73.1%, specificity 72%) and AHI ≥ 22.4 (sensitivity 79.5%, specificity 79.3%) as optimal cut-offs for predicting repolarization heterogeneity. SII, QTd, and TPEI were significantly associated with ventricular arrhythmias (*p* < 0.05). Patients with moderate-to-severe OSAS (AHI ≥ 15) had higher rates of ventricular tachyarrhythmias (17.8% vs. 5.7%, *p* = 0.03) and sudden cardiac death (9.3% vs. 1.9%, *p* = 0.05). *Conclusions*: Elevated SII and AHI are independent predictors of myocardial repolarization heterogeneity in patients with HF and OSAS, contributing to increased arrhythmic risk. These findings highlight the potential use of SII and AHI as accessible biomarkers for risk stratification, particularly in patients with a preserved ejection fraction, and underscore the need for targeted interventions to mitigate inflammation and OSAS severity.

## 1. Introduction

Heart failure (HF) remains a leading cause of global morbidity and mortality, imposing substantial healthcare burdens, with prevalence rates continuing to rise in aging populations worldwide [1]. Concurrently, obstructive sleep apnea syndrome (OSAS) has emerged as a highly prevalent disorder, characterized by repetitive episodes of complete or partial upper airway obstruction during sleep, resulting in recurrent hypoxemia, hypercapnia, intrathoracic pressure fluctuations, and consequent sleep fragmentation with excessive daytime somnolence [2]. The coexistence of HF and OSAS creates a high-risk milieu for cardiovascular complications, including increased hospitalizations, arrhythmic events, and mortality, posing significant therapeutic challenges [3,4].

The pathophysiological mechanisms linking OSAS to cardiovascular disease are complex and multifactorial. Recurrent apneic events trigger a cascade of physiological responses including negative intrathoracic pressure, hypoxemia, hypercapnia, and the activation of peripheral chemoreceptors. These stimuli lead to enhanced sympathetic nervous system activity, resulting in sympathovagal imbalance and autonomic dysfunction [5]. The consequent elevation of catecholamine levels, particularly epinephrine and norepinephrine, further contributes to cardiovascular instability and arrhythmogenesis. Moreover, chronic low-grade inflammation is increasingly recognized as a central factor in the pathogenesis of both OSAS and heart failure [6].

QT dispersion (QTd), the frontal QRS-T angle, and T wave peak-to-end interval (TPEI) are key electrocardiographic markers of myocardial repolarization heterogeneity [7,8]. QTd reflects the spatial heterogeneity in ventricular repolarization, associated with increased arrhythmic risk, while the QRS-T angle and TPEI indicate spatial and transmural repolarization discordance, respectively, contributing to ventricular arrhythmia susceptibility [9,10,11].

Despite substantial advances in understanding the individual pathophysiology of OSAS and HF, and their recognized synergistic effects on cardiovascular outcomes, critical knowledge gaps persist regarding the specific role of chronic inflammation in modulating cardiac electrical stability in this high-risk population. Therefore, this study aims to investigate the impact of baseline systemic inflammation on electrical heterogeneity in patients suffering from both HF and OSAS. By doing so, we seek to provide novel insights into inflammation-mediated electrical instability and refine risk stratification approaches in this vulnerable cohort.

## 2. Materials and Methods

This retrospective study included 160 patients with a diagnosis of heart failure who underwent overnight polysomnography and were subsequently diagnosed with OSAS at the Sleep Laboratory of Trakya University Medical Faculty Hospital between 1 January 2018 and 1 November 2024.

Patients were included if they were aged 18 years or older, had a confirmed diagnosis of HF based on the guidelines for the diagnosis and treatment of acute and chronic heart failure, and had undergone polysomnography confirming OSAS with an apnea–hypopnea index (AHI) ≥ 5 events/h [12]. Additional inclusion criteria included stable clinical status for at least 4 weeks prior to enrollment and willingness to provide informed consent.

To reduce potential confounding effects on systemic inflammatory status, the following exclusion criteria were applied: presence of known systemic inflammatory diseases (e.g., rheumatoid arthritis, systemic lupus erythematosus, vasculitis), chronic autoimmune disorders, chronic infections (e.g., tuberculosis, hepatitis B or C, HIV), active infections at the time of the sleep study, a history of malignancy, advanced hepatic or renal failure, hematological disorders affecting immune parameters, and the chronic use of immunosuppressive or anti-inflammatory medications.

Sociodemographic characteristics, cardiovascular risk factors, and detailed medical histories were obtained through the hospital’s electronic database, archived patient records from the sleep laboratory, and supplementary telephone interviews with patients when necessary, including both verbal confirmation and reviews of any available documentation.

Routine laboratory parameters collected prior to polysomnography were recorded. Inflammatory status was assessed using C-reactive protein (CRP) levels and complete blood count-derived values. SII—a recognized marker of immune-inflammatory activity—was calculated as follows: neutrophil count × platelet count/lymphocyte count [13].

Transthoracic echocardiographic findings, obtained in accordance with contemporary guideline-based recommendations, were retrieved from the hospital database [14]. AHI values were recorded from polysomnography reports. Electrocardiographic (ECG) data acquired during the sleep study were analyzed to determine the longest and shortest QT intervals, and QTd was subsequently calculated. QT dispersion is defined as the difference between the maximum and minimum QT intervals measured across a standard 12-lead electrocardiogram [15]. The corrected QT interval (QTc) was calculated using Bazett’s formula (QTc = QT/√RR). TPEI was measured as the interval from the peak of the T wave to its end in precordial leads. The frontal QRS-T angle was derived from the 12-lead ECG as the absolute difference between the QRS axis and the T wave axis in the frontal plane. TPEI, frontal QRS-T angle and QTc measurements were performed on standard 12-lead ECGs obtained prior to polysomnography. ECG measurements were performed by two independent cardiologists blinded to clinical data. Discrepancies in any measurements were resolved by consensus.

Metabolic syndrome was defined according to the revised National Cholesterol Education Program Adult Treatment Panel III (NCEP ATP III) criteria, requiring the presence of three or more of the following components: (1) waist circumference > 102 cm in men or >88 cm in women, (2) triglycerides ≥ 150 mg/dL or drug treatment for elevated triglycerides, (3) HDL cholesterol < 40 mg/dL in men or <50 mg/dL in women or drug treatment for reduced HDL-C, (4) blood pressure ≥ 130/85 mmHg or antihypertensive drug treatment, and (5) fasting glucose ≥ 100 mg/dL or drug treatment for elevated glucose [16].

Obesity was classified according to body mass index (BMI) as follows: normal weight (18.5–24.9 kg/m^2^), overweight (25.0–29.9 kg/m^2^), Class I obesity (30.0–34.9 kg/m^2^), Class II obesity (35.0–39.9 kg/m^2^), and Class III obesity (≥40.0 kg/m^2^). Among obese participants (BMI ≥ 30 kg/m^2^), metabolically healthy obesity (MHO) was defined as the presence of ≤1 metabolic syndrome component in addition to an elevated waist circumference, while metabolically unhealthy obesity (MUO) was defined as the presence of ≥2 additional metabolic syndrome components. This classification approach was based on established criteria that consider the absence of major cardiometabolic risk factors despite the presence of obesity [17].

Patients were stratified into two groups based on QT dispersion: QTd < 40 ms, indicating normal electrical heterogeneity, and QTd ≥ 40 ms, indicating increased electrical heterogeneity. A cutoff of ≥40 ms was selected, consistent with cardiology literature recognizing QTd as a non-invasive marker of repolarization heterogeneity and arrhythmic risk [18,19,20]. Prior studies have shown that values above this threshold predict inducible ventricular tachycardia in heart failure with 88% sensitivity and 57% specificity, and correlate with an increased risk of sudden cardiac death in various cardiovascular populations. [21]. For statistical analysis, electrical heterogeneity was defined based on QT dispersion (QTd ≥ 40 ms), consistent with its established role as a non-invasive marker of repolarization heterogeneity and arrhythmic risk in the study cohort. However, the frontal QRS-T angle and TPEI were also analyzed to provide a comprehensive evaluation of repolarization heterogeneity, as these parameters collectively capture spatial and transmural repolarization abnormalities [22,23]. To explore predictors of ventricular repolarization heterogeneity, ROC analysis was performed. Notably, ROC analysis for the SII was restricted to patients with QTc intervals below 500 ms in order to minimize the confounding influence of extreme QT prolongation on the assessment of repolarization heterogeneity [24]. In addition, patients were classified into two groups according to OSAS severity: mild (AHI 5–15 events/h) and moderate-to-severe (AHI ≥ 15 events/h), since a threshold of 15 events per hour is widely accepted in clinical practice as the criterion for recommending positive airway pressure therapy [5].

### Statistical Analysis

All statistical analyses were performed using the Statistical Package for the Social Sciences (SPSS), version 22.0 (SPSS Inc., Chicago, IL, USA). The normality of distribution for continuous variables was assessed using the Kolmogorov–Smirnov test. Continuous variables were expressed as mean ± standard deviation or median (minimum–maximum), depending on the distribution, while categorical variables were presented as frequencies and percentages.

Comparisons between groups were performed using the independent samples *t*-test for normally distributed variables and the Mann–Whitney *U* test for non-normally distributed variables. Associations between categorical variables were evaluated using the chi-square test or Fisher’s exact test, as appropriate.

To assess the potential predictors of QT dispersion, both univariate and multivariate logistic regression analyses were conducted. In addition, receiver operating characteristic (ROC) curve analysis was used to determine the sensitivity, specificity, area under the curve (AUC), and optimal cut-off values for the SII and AHI in predicting QT dispersion.

A *p*-value of <0.05 was considered statistically significant for all analyses.

## 3. Results

Participants included in the analysis had a mean age of 61.09 years (range: 32–85 years); 61.9% (*n* = 99) of the participants were male. The mean follow-up duration for the study population was 38 months.

Based on the QTd, patients were stratified into two groups: Group 1 (QTd < 40 ms; *n* = 82, 43 males) and Group 2 (QTd ≥ 40 ms; *n* = 78, 56 males). Additionally, based on left ventricular ejection fraction (LVEF), patients were categorized as follows: reduced EF (<40%; *n* = 13, 9 males), mildly reduced EF (40–49%; *n* = 23, 17 males), and preserved EF (≥50%; *n* = 124, 73 males). The baseline demographic characteristics, cardiac arrhythmia incidence, mortality outcomes and medications of the study population are presented in Table 1.

The baseline demographic and clinical characteristics differed significantly between the groups. Group 2 had a higher proportion of male patients, a greater prevalence of atrial fibrillation, and more frequent intracardiac device implantation. Heart failure phenotypes also varied, with Group 2 exhibiting higher rates of HFmrEF and lower rates of HFpEF. AHI values were significantly elevated in Group 2, reflecting more severe OSAS compared to Group 1. Furthermore, Group 2 demonstrated a higher incidence of ventricular tachyarrhythmias and sudden cardiac death. Other demographic and clinical variables, including age, comorbidities, and medication use, were similar between the groups.

Table 2 presents the laboratory findings of the study population, whereas Table 3 outlines the electrocardiographic and echocardiographic parameters.

The SII value was significantly higher in Group 2 compared to Group 1. An analysis of ECG parameters revealed that Group 2 exhibited a significantly increased frontal QRS-T angle and prolonged T wave peak-to-end interval, reflecting greater repolarization heterogeneity. Echocardiographic parameters, including the ejection fraction distribution, valvular regurgitation severity, and pulmonary artery pressure (PAP), were generally comparable between groups, with the exception of differences in ejection fraction categories.

The influence of clinical and laboratory variables on the development of QT dispersion was evaluated and is presented in Table 4. Among all variables analyzed, both SII and AHI were found to be statistically significant predictors of increased QT dispersion (*p* < 0.05).

In the univariate logistic regression analysis, higher SII values (*p* < 0.001) and elevated AHI (*p* < 0.001) were significantly associated with repolarization heterogeneity. Although diabetes mellitus, the white blood cell (WBC) count, and other clinical parameters showed trends toward association, they did not reach statistical significance in the univariate model.

Multivariate logistic regression analysis revealed that both SII (*p* = 0.01) and AHI (*p* < 0.001) remained independent predictors of increased QT dispersion after adjusting for potential confounders. In addition, diabetes mellitus was also independently associated with QT dispersion (OR: 2.538. 95% CI: 1.059–6.082. *p* = 0.04).

As shown in Table 5, univariate analysis revealed that QT dispersion, the T wave peak-to-end interval, and systemic immune-inflammation index (SII) were significantly associated with ventricular arrhythmias in patients with heart failure and obstructive sleep apnea syndrome (*p* < 0.05 for all). Additionally, the frontal QRS-T angle demonstrated a borderline association (*p* = 0.04). In the multivariate logistic regression model, which included variables with potential predictive value, only QT dispersion, the T wave peak-to-end interval, and SII remained as independent predictors of ventricular arrhythmia. (Additionally, as shown in Table 1, both ventricular arrhythmias and sudden cardiac death were evaluated, providing an overview of arrhythmic events in this cohort). These findings underscore the potential of electrophysiological markers and systemic inflammation in the risk stratification for arrhythmic events in this patient population.

As presented in Table 6, SII showed a significant positive correlation with QT dispersion, TPEI, and the frontal QRS-T angle. Additionally, SII was strongly correlated with the presence of ventricular arrhythmia. QTd and TPEI also correlated significantly with ventricular arrhythmia (r = 0.551 and r = 0.554, respectively; *p* < 0.001 for both). A weak correlation was noted between the frontal QRS-T angle and ventricular arrhythmia. No significant correlations were observed between QTc and any of the other parameters.

**Figure 1:**
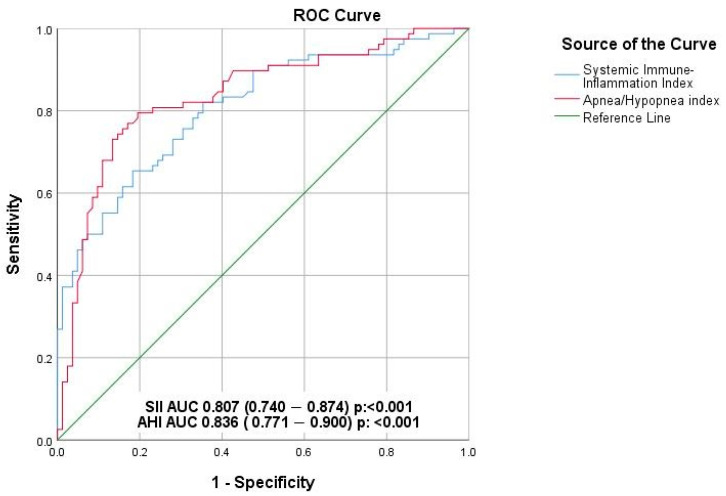
Effect of systemic immune inflammation index and apnea/hypopnea index on repolarization heterogeneity (ROC analysis).

ROC curve analysis was performed to determine the predictive value of both the AHI and the SII who are at risk of increased myocardial repolarization heterogeneity (Figure 1). An AHI cut-off value of 22.4 was found to predict QT dispersion with a sensitivity of 79.5% and a specificity of 79.3%, demonstrating a substantial AUC, indicative of a good discriminative ability. Similarly, an SII cut-off value of 625.4 predicted QT dispersion with a sensitivity of 73.1% and a specificity of 72%, reflecting predictive performance.

## 4. Discussion

Our study is the first to demonstrate that elevated SII and AHI, markers of chronic inflammation and OSAS severity, respectively, independently predict increased myocardial repolarization heterogeneity in patients with HF and OSAS, highlighting their synergistic role in promoting arrhythmogenic risk. Notably, this association was more prominent in patients without a reduced ejection fraction, suggesting that in the absence of advanced systolic dysfunction, the proarrhythmic impact of sleep apnea may be more apparent, potentially via mechanisms involving inflammation, oxidative stress, and autonomic imbalance.

QTd is an important electrophysiological marker that reflects spatial heterogeneity in ventricular repolarization. It serves as a surrogate indicator of increased arrhythmic vulnerability in the myocardium [7]. Early studies by Day et al. (1990) first established the prognostic significance of QT dispersion in post-myocardial infarction patients, demonstrating that QTd > 65 ms was associated with increased mortality [25]. This work was later refined by Malik and Batchvarov, who standardized measurement techniques and established the 40 ms threshold now widely accepted in clinical practice [26]. An elevated QTd independently predicts ventricular arrhythmias and sudden cardiac death in various cardiovascular conditions [27]. This spatial inhomogeneity in repolarization creates an electrophysiological milieu conducive to re-entrant arrhythmias, particularly in the setting of additional pro-arrhythmic triggers such as ischemia, electrolyte disturbances or autonomic imbalance [19]. While QTd has been widely used to assess repolarization heterogeneity, other complementary ECG parameters have also been proposed, including frontal QRS-T angle and TPEI. The frontal QRS-T angle represents the spatial relationship between ventricular depolarization and repolarization; an increase in this angle is considered an indicator of impaired electrical balance and increased arrhythmogenic risk. Additionally, the conversely, captures transmural repolarization gradients, reflecting the temporal dispersion of repolarization across the ventricular wall from epicardium to endocardium [9,10,11]. In our study, three complementary parameters—QT dispersion, frontal QRS-T angle, and TPEI—collectively provided a comprehensive assessment of ventricular electrical instability and all parameters were significantly elevated in patients with moderate to severe OSAS compared to those with mild disease, indicating increased ventricular electrical instability in this group.

The pathophysiological basis of these electrical abnormalities involves regional variations in ion channel expression, particularly L-type calcium channels, potassium channels (IKr, IKs, IK1), and sodium channels, which collectively determine action potential duration and morphology [19,28]. In heart failure, structural remodeling including myocardial fibrosis, cellular hypertrophy, and altered gap junction distribution creates zones of conduction delay and repolarization inhomogeneity [28]. Therefore, ECG parameters indicating ventricular repolarization are of particular clinical importance in this population, where ventricular arrhythmias constitute a significant proportion of sudden cardiac deaths [29]. Prior studies have shown that QTd correlates with disease severity, functional class, and the left ventricular ejection fraction, suggesting that electrical instability progresses alongside myocardial dysfunction [19,30]. The mechanistic basis of electrical heterogeneity in our study population appears to be multifactorial, involving both functional and structural components. Functional heterogeneity arises from regional differences in ion channel expression and calcium handling, while structural heterogeneity results from fibrotic replacement and altered myocardial architecture. The interaction between OSAS-induced intermittent hypoxia and chronic heart failure creates a unique pathophysiological environment where both mechanisms are amplified, potentially explaining why our patients exhibited more pronounced repolarization abnormalities compared to those with either condition alone. Our findings demonstrate that all three electrophysiological parameters were significantly elevated in patients with moderate-to-severe OSAS, suggesting a global electrical remodeling process rather than localized abnormalities. Furthermore, consistent with previous literature, our study observed that while ventricular repolarization heterogeneity is well documented in patients with a reduced ejection fraction, it also develops in patients with HFmrEF and HFpEF who have moderate-to-severe OSAS. Importantly, this repolarization heterogeneity in these patients was associated with an increased incidence of ventricular arrhythmias and cardiac arrest, highlighting its clinical relevance beyond traditional high-risk heart failure populations.

Previous studies in heart failure populations have identified several predictors of electrical heterogeneity, including the left ventricular ejection fraction, left atrial size, and myocardial fibrosis burden [31]. Galinier et al. further demonstrated that patients with HFrEF exhibited significantly higher QT dispersion compared to those with preserved function [32]. Turrini et al. also reported that QT dispersion > 40 ms predicted inducible ventricular tachycardia with 88% sensitivity in heart failure patients [21], while more recent investigations have highlighted the prognostic value of TPEI and the QRS-T angle in various cardiovascular conditions [9,10,11,21]. Our study extends these findings by showing that systemic inflammation measured by SII independently predicts electrical heterogeneity (correlated with all three markers), suggesting that inflammation is a unifying mechanism underlying diverse electrophysiological abnormalities. Notably, this relationship was most evident in patients with an elevated baseline systemic inflammatory status, a moderate and preserved ejection fraction, and concomitant OSAS.

The relationship between systemic inflammation and cardiac electrical stability involves complex molecular pathways that directly modulate myocardial electrophysiology. Elevated SII reflects a pro-inflammatory state characterized by increased neutrophil and platelet activation alongside lymphocyte suppression, creating a milieu conducive to cardiac electrical instability through multiple mechanisms [13,33,34].

Pro-inflammatory cytokines, particularly tumor necrosis factor-alpha (TNF-α), interleukin-6 (IL-6), and interleukin-1β (IL-1β), directly alter cardiac ion channel function and expression. TNF-α has been shown to reduce L-type calcium current (ICa,L) density and accelerate calcium channel inactivation, while simultaneously downregulating potassium currents including the transient outward current (Ito) and delayed rectifier currents (IKr, IKs) [11,28]. This dual effect prolongs the action potential duration while creating regional heterogeneity in repolarization timing. IL-6, elevated in both OSAS and heart failure, modulates sodium channel availability and reduces gap junction coupling through connexin-43 downregulation, contributing to conduction abnormalities and increased arrhythmogenic substrate [35,36]. Our results support the current mechanistic paradigm linking inflammation to electrical heterogeneity through ion channel modulation. Recent work by Aromolaran et al. demonstrated that TNF-α directly reduces IKr current density by 35% in human cardiomyocytes, providing a molecular basis for our observed SII-QTd correlation [37]. Similarly, Zamenina et al. showed that intermittent hypoxia alters calcium channel expression in animal models, supporting our finding of AHI as an independent predictor [38].

Oxidative stress, intrinsically linked to chronic inflammation, represents another crucial pathway linking SII elevation to electrical instability. Reactive oxygen species (ROS) generated during chronic inflammatory states directly damage cardiac ion channels, particularly affecting potassium channel function and calcium handling proteins including the ryanodine receptor (RyR2) and sarcoplasmic reticulum Ca^2+^-ATPase [39,40]. In OSAS patients, cyclical hypoxia–reoxygenation events amplify ROS production, creating sustained oxidative stress that progressively impairs cellular electrical function [39]. The correlation between SII and multiple repolarization parameters in our study (r = 0.417 for QTd, r = 0.425 for TPEI, r = 0.177 for QRS-T angle) suggests that a systemic inflammatory burden serves as a surrogate marker for the degree of oxidative stress-mediated ion channel dysfunction.

Furthermore, chronic inflammation promotes myocardial fibrosis through transforming growth factor-beta (TGF-β) and matrix metalloproteinase activation, creating structural substrates for electrical heterogeneity [41,42]. Fibrotic tissue creates zones of conduction block and slow conduction, establishing the anatomical basis for re-entrant arrhythmias. The finding that SII independently predicted both electrical heterogeneity and ventricular arrhythmias in our cohort supports this mechanistic pathway, suggesting that inflammatory markers may serve as early indicators of progressive electrical remodeling.

Our findings underscore the prognostic value of integrating systemic inflammation and OSAS severity with electrophysiological markers to enhance arrhythmic risk stratification in patients with HF and coexisting OSAS. Chronic systemic inflammation, as reflected by the SII, emerged as a key modulator of myocardial repolarization instability by disrupting cardiac ionic homeostasis and altering ion channel function [28,33]. SII showed significant positive correlations with key repolarization parameters—QTd, TPEI and frontal QRS-T angle—all of which were independently associated with ventricular arrhythmias in our cohort. Moreover, both SII and AHI were independent predictors of increased repolarization heterogeneity in multivariate analysis (*p* = 0.01 and *p* < 0.001, respectively), with optimal ROC-derived cut-off values of 625.4 for SII and 22.4 for AHI demonstrating good diagnostic performance [Figure 1]. Notably, patients with moderate-to-severe OSAS had significantly higher incidences of ventricular tachyarrhythmias (17.8% vs. 5.7%, *p* = 0.03) and sudden cardiac death (9.3% vs. 1.9%, *p* = 0.05), further emphasizing the clinical relevance of these parameters. Collectively, these results support a synergistic pathophysiological link between inflammation and repolarization abnormalities in promoting arrhythmogenic vulnerability, offering a framework for individualized risk assessment and the potential for targeted interventions such as CPAP therapy or anti-inflammatory strategies.

In patients with OSAS, recurring hypoxia–reoxygenation cycles, sympathetic overactivation, and systemic inflammation contribute to myocardial repolarization abnormalities [34,43]. Hypoxia alters potassium and calcium currents, while reoxygenation generates reactive oxygen species, promoting oxidative stress and membrane injury [39,41]. This cyclical damage promotes chronic cellular stress, electrical instability, and repolarization heterogeneity. Sympathetic surges during apneic events cause sustained autonomic imbalance and repolarization heterogeneity [40]. These processes, combined with upregulated pro-inflammatory cytokines (e.g., TNF-α, IL-6), foster myocardial fibrosis and electrical remodeling [35,42]. Mechanistically, chronic intermittent hypoxia activates nuclear factor-kappa B (NF-κB) signaling, enhancing pro-inflammatory gene expression and cytokine release [42]. Simultaneously, hypoxic episodes disrupt calcium homeostasis through endoplasmic reticulum stress and mitochondrial dysfunction, creating calcium overload conditions that predispose individuals to delayed afterdepolarizations and triggered activity [39,40]. Clinically, the link between OSAS and electrical heterogeneity was first demonstrated by Rossi et al., who reported significantly increased QT dispersion in OSAS patients compared to controls, although heart failure patients were not included [44]. Later, Voigt et al. showed that CPAP therapy reduced QT dispersion in OSAS patients by approximately 15% over 6 months [45]. Our study builds on these observations by demonstrating that AHI independently predicts electrical heterogeneity in the specific context of heart failure, with clinically actionable cut-off values (AHI ≥ 22.4). The cyclical nature of OSAS-related insults thus creates a progressive accumulation of electrical abnormalities, explaining the dose–response relationship we observed between AHI severity and repolarization parameters.

While our findings suggest that the SII is associated with electrical heterogeneity in patients with severe OSAS, the generalizability of SII as a predictor of electrical heterogeneity across all OSAS severities is limited. Previous studies have reported elevated inflammatory markers in OSAS, and our data confirm higher SII levels in patients with severe OSA compared to those with mild OSA. This variability in inflammatory profiles across OSAS severities suggests that SII may not uniformly predict electrical heterogeneity in all OSAS patients. Consequently, these results should be interpreted with caution, and further studies in larger, more diverse cohorts are needed to validate the predictive role of SII across the spectrum of OSAS severity

Subgroup analysis revealed that the impact of AHI on QTd was more pronounced in patients with heart failure with a moderate (HFmEF) and preserved ejection fraction (HFpEF) compared to those with a reduced ejection fraction (HFrEF). This observation addresses a notable gap in the literature, as previous studies, such as Safabakhsh et al., suggested that HFpEF patients might have distinct arrhythmic risk profiles compared to HFrEF but lacked mechanistic insights [46]. The heightened vulnerability in HFpEF may stem from multiple pathophysiological mechanisms unique to this phenotype, including coronary microvascular dysfunction, diastolic stiffness, and impaired baroreceptor sensitivity, which exacerbate repolarization abnormalities during apneic episodes [36,47,48,49]. Chronic intermittent hypoxia in OSAS promotes systemic inflammation, oxidative stress, and subclinical myocardial fibrosis, altering ion channel expression and conduction properties to amplify repolarization heterogeneity [50,51,52]. Additionally, impaired baroreceptor sensitivity in moderate and preserved ejection fractions may exacerbate autonomic imbalance, further increasing myocardial electrical instability during apneic events [53,54]. In contrast to HFrEF, where compensatory mechanisms may mitigate some electrophysiological perturbations, the preserved systolic function in HFpEF appears to render the myocardium more susceptible to inflammation- and hypoxia-mediated repolarization abnormalities, potentially explaining the stronger AHI-QTd association in this subgroup [55]. These findings underscore the critical interplay between OSAS severity, systemic inflammation, and electrophysiological changes in HFpEF, highlighting the need for targeted risk stratification in this population.

Obesity, a common comorbidity in HF and OSAS, amplifies arrhythmic vulnerability by promoting systemic inflammation, as reflected by SII [56,57]. As shown in Table 1, the prevalence of obesity was similar between the two groups, indicating that the elevated inflammatory burden observed in group 2 cannot be explained by differences in adiposity. Furthermore, in light of these findings, obesity does not appear to act as an independent predictor of ventricular arrhythmias in this patient population. This is further supported by the multivariate analysis results presented in Table 5, where obesity did not retain statistical significance, suggesting that other factors such as OSAS severity and systemic inflammation exert a more direct influence on myocardial electrical instability.

In addition to the molecular and electrophysiological mechanisms linking systemic inflammation to myocardial repolarization heterogeneity, the cardiac lymphatic system may play a pivotal role in modulating structural and electrical dispersion in patients with HF and OSAS. Chronic inflammation, as reflected by elevated CRP levels, has been shown to impair lymphatic function by reducing lymphangion amplitude and frequency, leading to compromised lymphatic drainage and subsequent interstitial edema [58]. The cardiac lymphatic system is critical for removing metabolic debris and interstitial fluid from the myocardium, maintaining tissue homeostasis and structural integrity. Impaired lymphatic drainage, driven by systemic inflammation, promotes myocardial edema, which alters the myocardial architecture and creates zones of electrical dispersion conducive to arrhythmias.

Obstructive sleep apnea further exacerbates lymphatic dysfunction through elevated right heart pressures during apneic episodes, which impede lymphatic flow into the central circulation [59]. These elevated pressures, resulting from recurrent hypoxia and increased intrathoracic pressure swings, disrupt the normal clearance of interstitial fluid, amplifying myocardial edema and structural remodeling. This process contributes to the formation of heterogeneous conduction pathways, increasing the substrate for re-entrant arrhythmias. The interplay between systemic inflammation and OSAS-induced lymphatic dysfunction thus represents a novel mechanistic pathway linking elevated SII and AHI to increased repolarization heterogeneity and arrhythmic risk.

Therapeutic strategies targeting lymphatic function and pulmonary hypertension may offer potential benefits in this population. Interventions such as continuous positive airway pressure (CPAP) therapy, which mitigates apneic episodes and reduces right heart pressures, could enhance lymphatic drainage and decrease myocardial edema. Additionally, anti-inflammatory therapies aimed at reducing CRP and other pro-inflammatory mediators may restore lymphatic contractility, further alleviating structural and electrical abnormalities. Moreover, the identification of lymphatic dysfunction as a potential therapeutic target opens new avenues for intervention. Future research should investigate whether targeted lymphatic drainage techniques or pharmacological agents that enhance lymphatic contractility could provide additional benefits in this patient population, potentially serving as adjunctive therapies to conventional heart failure and OSAS management. These approaches warrant further investigation to determine their efficacy in reducing arrhythmic risk in patients with HF and OSAS.

Our findings suggest that elevated SII and AHI values may serve as accessible and clinically meaningful indicators for identifying patients at an increased risk of ventricular arrhythmias within the HF and OSAS population. In particular, these markers can aid in individualizing pharmacotherapeutic strategies—especially in scenarios where the use of QT-prolonging or proarrhythmic medications is unavoidable. Recognizing elevated SII as a potential red flag may prompt clinicians to consider safer alternatives, optimize electrolyte balance, and systematically evaluate the cumulative burden of arrhythmogenic ECG parameters. Such an approach could enhance arrhythmic risk mitigation in routine care. Furthermore, these findings lay the groundwork for future investigations into whether interventions aimed at reducing systemic inflammation—such as CPAP or targeted anti-inflammatory therapies—can favorably modulate SII levels and decrease the associated electrophysiological risk in this high-risk population.

This study has several limitations that warrant consideration. First, its retrospective, single-center design and relatively modest sample size may limit the generalizability of the findings to broader patient populations. Furthermore, the absence of an independent external validation cohort represents a significant limitation for our proposed novel clinical markers. The lack of cross-validation techniques or replication in an independent dataset constrains our ability to confirm the robustness and generalizability of the SII cutoff values and predictive models presented. Second, although we observed a significant association between systemic inflammation and electrical heterogeneity, the lack of invasive electrophysiological assessments precludes a direct evaluation of the arrhythmogenic substrate underlying this relationship. Additionally, the potential influence of unmeasured confounding variables could not be fully excluded.

Another important limitation is the heterogeneity in metabolic phenotypes within our patient population, particularly regarding obesity-related cardiovascular risk stratification. While we collected comprehensive metabolic syndrome components and implemented obesity classification criteria, the complex metabolic heterogeneity among obese patients may have influenced our inflammatory and electrophysiological associations. Additionally, we lacked a detailed assessment of insulin resistance indices (such as HOMA-IR), adiponectin levels, and other adipokines that could provide deeper insights into the metabolic-inflammatory axis. The absence of body composition analysis (such as visceral versus subcutaneous fat distribution) represents another limitation, as different adipose tissue compartments exhibit distinct inflammatory profiles that may differentially impact cardiac electrophysiology [60]. Prospective, multicenter studies with larger sample sizes, independent validation cohorts, and detailed metabolic and electrophysiological characterization are needed to validate our findings and further clarify the clinical significance of repolarization heterogeneity in this high-risk patient population.

Before concluding, it is important to summarize the key findings and highlight areas requiring further exploration. Our study demonstrates that both elevated SII and AHI are independently associated with increased myocardial repolarization heterogeneity in patients with heart failure and coexisting obstructive sleep apnea. Notably, these associations were more pronounced in patients with a preserved ejection fraction, suggesting a unique vulnerability to inflammation- and hypoxia-mediated electrophysiological alterations in this subgroup. These findings underscore the potential of SII as a simple and cost-effective biomarker for identifying patients at heightened arrhythmic risk, particularly in resource-limited settings where advanced monitoring is not always feasible.

## 5. Conclusions

This study demonstrates that elevated SII and AHI are independently associated with increased myocardial repolarization heterogeneity, a non-invasive marker of myocardial repolarization heterogeneity in patients with concomitant HF and OSAS. These findings suggest that chronic low-grade systemic inflammation may contribute to the development of electrical inhomogeneity within the myocardium, potentially increasing arrhythmogenic risk in this vulnerable population. Furthermore, the identification of SII and AHI as predictors of increased myocardial repolarization heterogeneity highlights their potential utility as accessible and cost-effective markers in risk stratification. Future large-scale and longitudinal studies incorporating advanced electrophysiological and imaging modalities are warranted to validate these associations and explore their prognostic implications in clinical practice.

## Figures and Tables

**Table 1:** Demographic characteristics, cardiac arrhythmia incidence, mortality outcomes and medications of the study population.

Characteristics		Group 1 (*n* = 82)	Group 2 (*n* = 78)	*p*-Value
Age (years)		61.4 ± 10.5	60.7 ± 12.7	0.68
Male gender, *n* (%)		43 (52.4)	56 (71.8)	0.01
Hypertension, *n* (%)		61 (74.4)	60 (76.9)	0.07
Diabetes Mellitus, *n* (%)		24 (29.3)	31 (39.7)	0.16
Atriyal fibrilasyon, *n* (%)		5 (6.1)	14 (17.9)	0.02
Stroke, *n* (%)		6 (7.3)	5 (6.4)	0.82
Coronary artery disease, *n* (%)		43 (52.4)	43 (55.1)	0.73
Intracardiac device, *n* (%)		1 (1.2)	7 (9)	0.02
Smoking, *n* (%)		19 (23.2)	12 (15.4)	0.21
HFrEF (LVEF ≤ 40%), *n* (%)		6 a (7.3)	7 a (9)	
HFmrEF (LVEF 41–49%), *n* (%)		6 a (7.3)	17 b (21.8)	0.03
HFpEF (LVEF ≥ 50%), *n* (%)		70 a (85.4)	54 b (69.2)	
BMI, kg/m^2^		34.3 ± 3.4	34.9 ± 2.5	0.17
	Overweight, *n* (%)	10 a (12.2)	0 b (0)	0.004
	Class 1 Obesity, *n* (%)	39 a (47.6)	36 a (46.2)	
	Class 2 Obesity, *n* (%)	33 a (40.2)	42 a (53.8)	
Obesity Classification				
Healthy Obesity		27 (37.5)	24 (30.8)	0.38
Unhealthy Obesity		45 (62.5)	54 (69.2)	
Metabolic Syndrome, *n* (%)		46(86.8)	80(74.8)	0.33
Apnea-Hypopnea index				
	<15 events/h	45 a (54.9)	8 b (10.3)	<0.001
	≥15 events/h	37 a (45.1)	70 b (89.7)	
Fasting Glucose, (mg/dL)		116.5 (92–201)	116 (78–135)	0.06
HDL, (mg/dL)		41.6 ± 6.5	43.1 ± 6.4	0.15
Triglycerides, (mg/dL)		158.7 ± 13.7	158.9 ± 15.5	0.93
Systolic blood pressure, (mmHg)		132.9 ± 8.8	130.9 ± 8.6	0.15
Dystolic blood pressure, (mmHg)		81.1 ± 5.3	80 ± 4.5	0.14
Waist circumference		107.2 ± 8.3	109.7 ± 8.3	0.06
Ventricular tachyarrhythmia, *n* (%)		0 (0)	22 (28.2)	<0.001
Sudden cardiac death, *n* (%)		0 (0)	11 (14.1)	<0.001
Beta blocker, *n* (%)		42 (51.2)	45 (57.7)	0.41
SGLT2 inhibitors, *n* (%)		21 (25.6)	28 (35.9)	0.15
RAS blocker, *n* (%)				
	Not using	19 (23.2)	30 (38.5)	0.08
	ACEi/ARB	60 (73.2)	47 (60.3)	
	Sacubitril valsartan	3 (3.7)	1 (1.3)	
MRA, *n* (%)		14 (17.1)	12 (15.4)	0.77
Loop Diüretics, *n* (%)		39 (47.6)	33 (42.3)	0.5
Ranelozin, *n* (%)		18 (22)	23 (29.5)	0.27
Statin, *n* (%)		36 (43.9)	46 (59)	0.06

Values are mean ± SD. *n* (%). Continuous variables (mean ± SD) calculated using the independent *t*-tests. [*n* (%)] calculated using chi-square tests. a-b = There is no significant difference between groups with the same letter.

**Table 2:** Laboratory findings of the study populations.

Variable		Group 1 (*n* = 82)	Group 2 (*n* = 78)	*p*-Value
WBC count (10^3^/µL)	EF < 40%	10.7 ± 2.1	9.2 ± 1.8	0.59
	EF 40–50%	9.4 ± 3.5	8.6 ± 3	
	EF > 50%	8.3 ± 2.2	8.8 ± 2.3	
Hemoglobin. (mg/dL)	EF < 40%	13.8 (11.1–16)	13.2 (10.3–16.4)	0.75
	EF 40–50%	14.7 (13.9–15.1)	13.3 (11.3–17)	
	EF > 50%	13.7 (8.1–17.3)	13.9 (8.5–17)	
Creatinine. (mg/dL)	EF < 40%	0.8 (0.7–1.1)	0.7 (0.7–0.9)	0.47
	EF 40–50%	0.9 (0.8–1)	1 (0.7–1.6)	
	EF > 50%	0.9 (0.5–2.2)	0.9 (0.5–10.5)	
GFR. (mL/min/1.79 m^2^)	EF < 40%	90.3 (79.5–94.7)	95.7 (87.7–116.8)	0.87
	EF 40–50%	90.6 (67.3–102.3)	86.4 (34.3–102.9)	
	EF > 50%	93.3 (11.5–114.2)	90.9 (4.8–126.7)	
CRP. (mg/dL)	EF < 40%	8.3 (4.4–50)	10 (0.2–21.7)	0.72
	EF 40–50%	2.3 (0.3–4.2)	5.2 (0.2–83.5)	
	EF > 50%	3.1 (0.1–32.2)	3 (0.2–19.7)	
ALT. (µ/L)	EF < 40%	16 (13–35)	19 (12–50)	0.36
	EF 40–50%	20 (17–46)	21 (10–42)	
	EF > 50%	20 (9–57)	20.5 (9–63)	
AST. (µ/L)	EF < 40%	18.5 (13–29)	17 (12–59)	0.79
	EF 40–50%	23.5 (14–31)	22 (15–40)	
	EF > 50%	20.5 (8–46)	19.5 (9–71)	
Neutrophil count. (10^3^/µL)	EF < 40%	6.2 (2.3–7.8)	5.4 (3.9–8.2)	0.005
	EF 40–50%	4.8 (2.2–11.3)	5.2 (2.2–12.6)	
	EF > 50%	4.7 (2.1–9.9)	5.5 (3.5–9.1)	
Platelet count. (10^3^/µL)	EF < 40%	254.2 ± 95.2	279 ± 71.2	0.001
	EF 40–50%	233.8 ± 29.2	260.6 ± 62.7	
	EF > 50%	251.3 ± 57.8	286.3 ± 56.4	
Lymphocyte count. (10^3^/µL)	EF < 40%	2.4 (1.1–3.1)	2.1 (1.5–3.1)	0.09
	EF 40–50%	3.2 (1.7–4.5)	2.1 (0.7–3.5)	
	EF > 50%	2.4 (1–22.6)	2.3 (0.7–5.4)	
SII	EF < 40%	578.1 (349.2–951.8)	756 (294.7–1126.7)	<0.001
	EF 40–50%	278.8 (218.3–1229.4)	621 (198.2–2633.4)	
	EF > 50%	476.7 (127.1–1085.2)	691.5 (271.1–2747.7)	

Values are mean ± SD. median (min–max). “mean ± SD” calculated using the independent *t*-tests. “median (min–max)” calculated using the Mann–Whitney U tests. LVEF subgroups are presented for descriptive purposes only. No statistical comparisons were stratified by LVEF in this table.

**Table 3:** Echocardiographic and ECG findings of the study population.

Variable		Group 1 (*n* = 82)	Group 2 (*n* = 78)	*p*-Value
EF. *n* (%)	EF < 40%	6 a (7.3)	7 a (9)	0.03
	EF 40–50%	6 a (7.3)	17 b (21.8)	
	EF > 50%	70 a (85.4)	54 b (69.2)	
Mitral regurgitation. *n* (%)	Severe	1 (1.2)	2 (2.6)	0.85
	Moderate	11 (13.4)	13 (16.7)	
	Mild	46 (56.1)	41 (52.6)	
	Normal	24 (29.3)	22 (28.2)	
Aortic regurgitation. *n* (%)	Severe	0 (0.0)	0 (0.0)	0.68
	Moderate	3 (3.7)	5 (6.4)	
	Mild	37 (45.1)	32 (41)	
	Normal	42 (51.2)	41 (52.6)	
Tricuspid regurgitation. *n* (%)	Severe	2 (2.4)	4 (5.1)	0.6
	Moderate	15 (18.3)	13 (16.7)	
	Mild	50 (61)	50 (64.1)	
	Normal	15 (18.3)	10 (12.8)	
QT Dispersion	EF < 40%	31 (24–33)	57 (43–77)	<0.001
	EF 40–50%	28.5 (18–35)	54 (40–76)	
	EF > 50%	28 (10–39)	56.5 (40–82)	
Longest QT	EF < 40%	441 (370–502)	506 (482–532)	<0.001
	EF 40–50%	430 (380–490)	472 (395–545)	
	EF > 50%	405.5 (358–510)	476 (398–540)	
Shortest QT	EF < 40%	410 (346–470)	457 (405–475)	<0.001
	EF 40–50%	405 (350–458)	420 (350–490)	
	EF > 50%	377 (235–475)	420 (346–496)	
PAB	EF < 40%	45.5 (37–47)	40 (20–48)	0.32
	EF 40–50%	35 (20–53)	32 (20–70)	
	EF > 50%	24.5 (20–80)	25 (20–70)	
	EF < 40%	424.8 ± 5.8	412.4 ± 11.5	0.45
QTc interval	EF 40–50%	430.4 ± 23.9	418 ± 15.4	
	EF > 50%	417.5 ± 15.8	422.9 ± 17.8	
Frontal QRS-T Angle	EF < 40%	55.1 ± 25	70.8 ± 28.8	0.03
	EF 40–50%	64.6 ± 15.9	62.7 ± 24.9	
	EF > 50%	64.6 ± 27	69.9 ± 30.3	
T wave peak-to end Interval	EF < 40%	85 ± 10.5	82.5 ± 19.2	0.02
	EF 40–50%	83.1 ± 14.2	89 ± 13.3	
	EF > 50%	79.1 ± 11.9	84.6 ± 17.3	

Values are mean *n* (%). median (min–max). [*n* (%)] calculated using chi-square tests. [median (min-max)] calculated using Mann–Whitney U tests.). a-b = There is no significant difference between groups with the same letter. LVEF subgroups are presented for descriptive purposes only. No statistical comparisons were stratified by LVEF in this table.

**Table 4:** Univariate and multivariate predictors of electrical heterogeneity in patients with heart failure and obstructive sleep apnea syndrome.

	Univariate Analysis			Multivariate Analysis		
	Odds Ratio	(95% C.I. for Odds Ratio)	*p* *	Odds Ratio	(95% C.I. for Odds Ratio)	*p* *
WBC	1.037	(0.909–1.183)	0.59	0.823	(0.666–1.018)	0.07
SII	1.001	(1–1.002)	<0.001	1.002	(1–1.003)	0.01
GFR	1.001	(0.987–1.015)	0.86	1.001	(0.977–1.026)	0.93
Apnea/Hypopnea index	1.059	(1.04–1.079)	<0.001	1.066	(1.044–1.088)	<0.001
Age	0.994	(0.968–1.021)	0.68	0.997	(0.951–1.046)	0.91
Hypertension	1.148	(0.557–2.366)	0.71	0.696	(0.253–1.917)	0.48
Diabetes Mellitus	1.594	(0.826–3.075)	0.16	2.538	(1.059–6.082)	0.04
PAB	1.015	(0.99–1.041)	0.24	1.024	(0.99–1.06)	0.17

* Calculated using the enter method. Abbreviation: GFR, Glomerular filtration rate; SII. Systemic Immune-Inflammation Index; PAB, Pulmonary artery hypertension.

**Table 5:** Univariate and multivariate predictors of ventricular arrhythmia in patients with heart failure and obstructive sleep apnea syndrome.

	Univariate Analysis			Multivariate Analysis		
	Odds Ratio	(95% C.I. for Odds Ratio)	*p* *	Odds Ratio	(95% C.I. for Odds Ratio)	*p* *
Apnea/Hypopnea index	1.014	(0.999–1.03)	0.07	0.883	(0.776–1.004)	0.057
Body Mass Index	1.111	(0.952–1.295)	0.18	1.453	(0.744–2.836)	0.27
QTc	0.994	(0.967–1.021)	0.64	1.03	(0.923–1.15)	0.59
QT Dispersion	1.146	(1.087–1.208)	<0.001	1.694	(1.107–2.592)	0.02
Frontal QRS-T Angle	1.017	(1.001–1.033)	0.04	0.984	(0.921–1.05)	0.62
T wave peak-to end Interval	1.224	(1.125–1.333)	<0.001	1.306	(1.036–1.646)	0.02
LVEF	0.981	(0.936–1.027)	0.4	1.218	(0.959–1.547)	0.11
SII	1.006	(1.004–1.009)	<0.001	1.004	(1.001–1.008)	0.01

* Calculated using the enter method. Abbreviation: LVEF, Left ventricular ejection fraction; QTc, corrected QT.

**Table 6:** Correlation analysis of systemic immune inflammation index and ECG markers of electrical heterogeneity in patients with ventricular arrhythmia.

	Ventricular Arrhythmia		SII		QT Dispersion		Frontal QRS-T Angle		TPEI		QTc	
	r	*p*	r	*p*	r	*p*	r	*p*	r	*p*	r	*p*
SII	0.673 **	<0.001										
QT Dispersion	0.551 **	<0.001	0.417 **	<0.001								
Frontal QRS-T Angle	0.169 *	0.03	0.177 *	0.03	0.15	0.06						
TPEI	0.554 **	<0.001	0.425 **	<0.001	0.275 **	<0.001	0.166 *	0.04				
QTc	0.37	0.64	<0.001	0.97	0.02	0.79	0.02	0.79	0.12	0.13		

** Correlation is significant at the 0.01 level (2-tailed), * Correlation is significant at the 0.05 level. Abbreviation: QTc, corrected QT; SII, Systemic Immune-Inflammation Index; TPEI, T wave peak-to end Interval.

## Data Availability

The datasets used and/or analyzed during this study are available. from the corresponding author on reasonable request.
